# Origins of Spatial Working Memory Deficits in Schizophrenia: An Event-Related fMRI and Near-Infrared Spectroscopy Study

**DOI:** 10.1371/journal.pone.0001760

**Published:** 2008-03-12

**Authors:** Junghee Lee, Bradley S. Folley, John Gore, Sohee Park

**Affiliations:** 1 Department of Psychology and the Center for Integrative and Cognitive Neuroscience, Vanderbilt University, Nashville, Tennessee, United States of America; 2 Vanderbilt University Kennedy Center, Nashville, Tennessee, United States of America; 3 Institute of Imaging Science, Department of Radiology, Vanderbilt University, Nashville, Tennessee, United States of America; 4 Department of Psychiatry, Vanderbilt University School of Medicine, Nashville Tennessee, United States of America; Claremont Graduate University, United States of America

## Abstract

Abnormal prefrontal functioning plays a central role in the working memory (WM) deficits of schizophrenic patients, but the nature of the relationship between WM and prefrontal activation remains undetermined. Using two functional neuroimaging methods, we investigated the neural correlates of remembering and forgetting in schizophrenic and healthy participants. We focused on the brain activation during WM maintenance phase with event-related functional magnetic resonance imaging (fMRI). We also examined oxygenated hemoglobin changes in relation to memory performance with the near-infrared spectroscopy (NIRS) using the same spatial WM task. Distinct types of correct and error trials were segregated for analysis. fMRI data indicated that prefrontal activation was increased during WM maintenance on correct trials in both schizophrenic and healthy subjects. However, a significant difference was observed in the functional asymmetry of frontal activation pattern. Healthy subjects showed increased activation in the right frontal, temporal and cingulate regions. Schizophrenic patients showed greater activation compared with control subjects in left frontal, temporal and parietal regions as well as in right frontal regions. We also observed increased ‘false memory’ errors in schizophrenic patients, associated with increased prefrontal activation and resembling the activation pattern observed on the correct trials. NIRS data replicated the fMRI results. Thus, increased frontal activity was correlated with the accuracy of WM in both healthy control and schizophrenic participants. The major difference between the two groups concerned functional asymmetry; healthy subjects recruited right frontal regions during spatial WM maintenance whereas schizophrenic subjects recruited a wider network in both hemispheres to achieve the same level of memory performance. Increased “false memory” errors and accompanying bilateral prefrontal activation in schizophrenia suggest that the etiology of memory errors must be considered when comparing group performances. Finally, the concordance of fMRI and NIRS data supports NIRS as an alternative functional neuroimaging method for psychiatric research.

## Introduction

Working memory is a limited-capacity, active short-term memory system that guides and controls behavior in context. The working memory deficit in schizophrenia, first reported in the early 90s [1] has become an important cornerstone in understanding the pathophysiology of this disorder. Our earlier studies as well as a new meta-analysis [2] indicate that stable and reliable working memory deficits are observed in schizophrenic patients across diverse paradigms, methods and techniques. In addition, individuals at risk for schizophrenia, such as healthy, unmedicated first-degree relatives of schizophrenic subjects [3]–[5] and psychometrically-ascertained schizotypal subjects also show working memory deficits [6]–[9]. In schizophrenic subjects, working memory deficit is permanent and stable even when their clinical symptoms are largely in remission [10], [11]. The persistent and trait-like nature of working memory deficit in schizophrenic patients and in healthy first-degree relatives suggests that it may be an endophenotypic marker for schizophrenia [3], [12], [13], and hence a potential aid to discovering the genetic basis of this disorder. Moreover, working memory deficit may be central to cognitive deficits [14], which predict functional outcome better than do clinical symptoms [15]–[17] and it is associated with poor social functioning in schizophrenia [7], [8]. Not surprisingly, working memory deficits have become a major therapeutic target for pharmacological and behavioral treatment strategies. Thus, it is important to specify the origins and consequences of the deficit but we do not know yet know much about the reasons for the deficit.

Much is known about the role of the dorsolateral prefrontal cortex (DLPFC) in working memory and its regulation of higher cognitive functions in non-human primates [18], [19]. In nonhuman primates, working memory has been studied extensively with the delayed-response task since 1930s [20]. A prototypical delayed-response task involves presentation of a stimulus, followed by a short delay and the subsequent presentation of response choices. The ability to perform a delayed-response task is compromised by lesions in the DLPFC [18], [19]. The principal sulcus (PS, Area 46) is thought to mediate spatial working memory, because neurons in the principal sulcus are involved in maintenance of spatial information over time [21]–[23]. For example, when a saccadic eye movement to a target is delayed, the neurons in the principal sulcus increase and maintain firing during the delay period, but as soon as the response is made, the firing rate decreases rapidly to the baseline level. These neurophysiological data also indicate that a robust increase in the prefrontal neuronal activity during the delay period is correlated with the accuracy of working memory [21]–[23].

In healthy humans, better working memory performance is correlated with increased prefrontal activation [24]–[28]. In contrast, the relationship between working memory accuracy and prefrontal activation in schizophrenic patients is not yet clearly understood. Many neuroimaging studies of working memory have demonstrated *task-related hypofrontality* in schizophrenic patients [29]–[31]. However, there are also studies showing evidence of increased activation in the prefrontal cortex (‘hyperfrontality’) in schizophrenia patients [32], [33]. Thus a simple hypofrontality hypothesis cannot account for working memory deficits in schizophrenia. To date, hypofrontality, hyperfrontality, reduced asymmetry, “inefficiency” and other factors have been proposed to account for working memory deficits [34]–[36], but how these factors influence the degree or the direction of prefrontal activations with respect to working memory performance is poorly understood. These results suggest that the task demands and performance must be considered in evaluating the cortical activation patterns of in schizophrenic patients and healthy controls. We also need to better understand what “activation” means in neuroimaging studies. Activating a system or network may not necessarily correlate with the behavioral performance. Inactivation may be the result of many factors. One reason may be a failure to engage the network that is needed to perform a certain function; the hypofrontality hypothesis of schizophrenia assumes this. However, neural activation may also reflect the degree of expertise. The neural network(s) involved in a task when a subject is a novice shifts to a different engagement of other networks as the subject becomes more skilled [37]. Thus, inactivity of a network may signify automaticity and expertise rather than failure. Inactivation of a certain region might also mean that a different system is engaged to perform the same task (different strategy) or that even if the same brain regions are activated for a particular task, they may come “on-line” at different times [35]. Thus, we need to better understand what activation patterns signify in functional neuroimaging studies when different diagnostic groups (who may use different strategies) are being compared.

The present study planned to investigate whether working memory performance in schizophrenia is tightly coupled with prefrontal activation, as it is generally observed in healthy individuals [28], [38]. Previous functional neuroimaging studies of working memory comparing schizophrenia patients and healthy controls do not provide adequate information concerning the degree of association between working memory performance and prefrontal cortical activation in schizophrenia patients because the control and patient groups were not matched for performance accuracy [e.g.], [29], [31,32]. Other studies controlled for this difference in performance by matching overall working memory accuracy between the two groups and still found reduced dorsolateral prefrontal activation in schizophrenia patients [30], [39]. However, matching overall accuracy alone may not be sufficient if different types of memory errors are elicited via different mechanisms in schizophrenia patients. We have argued elsewhere [3], [6], [13] that there are distinct processes that lead to working memory errors (e.g., problems in encoding, maintenance or inhibition). For example, working memory errors may arise from a failure to encode the target or from losing internal representations during maintenance; such error trials should correlate with reduced or absent prefrontal activation because active maintenance did not occur. In contrast, a very different type of errors will be elicited if participants encode a non-target object or location (i.e., incorrect stimulus) and maintain this representation successfully during the delay. This is akin to a “false memory” because the incorrect stimulus has been encoded and maintained, producing incorrect behavioral response. However, since its internal representation was maintained during the delay, this type of error should result in increased prefrontal activation. On such false memory trials, subjects maintain an internal representation, albeit a wrong one, and they are also likely to be confident of their responses they make based on this representation maintained in working memory. Therefore, to understand the relationship between working memory performance and cortical activation patterns, finer analyses of behavioral performance and error types in schizophrenia patients are needed and it is critical to differentiate distinct error types as well as examining correct and error trials separately. However, different types of errors have not been distinguished in functional neuroimaging studies of working memory.

**Figure 1:**
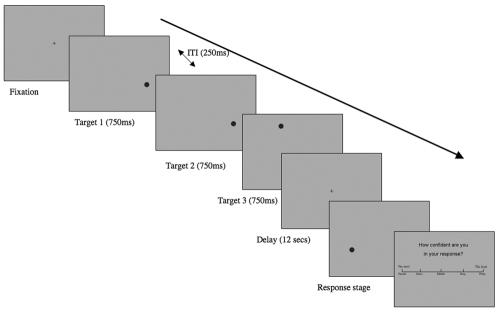
A schematic diagram of the spatial working memory (WM) task. After the presentation of a fixation point (1000 ms), three targets appeared one after the other for 750 ms each with an interstimulus interval of 250 ms. A delay period of 12 s followed. Then a probe was presented. Participants were asked to decide whether the probe was located in one of the target locations or not. After they made a response, they were asked to rate their confidence level for the response that they had just made.

The main goal of this study was to elucidate the neural correlates of working memory in schizophrenia by focusing on cortical activation during correct and error trials with two functional imaging experiments. In Experiment 1, we compared cortical activation in schizophrenia patients and normal controls on a spatial delayed response task (see Figure 1) using fMRI, focusing on correct vs. error trials. In a spatial delayed response task, participants were asked to remember locations of targets after a delay period of 12 seconds, which allowed us to separate correct vs. error trials based on the accuracy of their performance. To differentiate different types of errors, we asked participants to rate the confidence of their response. Correct and error responses were further divided according to the confidence ratings. Errors with very high confidence ratings suggest that subjects were certain of remembering *something* albeit an inappropriate one (e.g., false memory errors). On the other hand, errors with very low confidence ratings would indicate that these were true memory errors.

The second goal of this study was to test the less invasive near-infrared spectroscopy (NIRS) as a viable alternative to fMRI using the same task employed in Experiment 1. fMRI has become the standard brain imaging method to examine the neural correlates of cognitive function, but the MRI environment can lead to difficulties in recruiting psychiatric patients because of stringent exclusion criteria. Moreover, many patients exceed the safety weight limit of the scanner, in part due to atypical antipsychotic medication [40]. Thus, it is possible that patients who participate in fMRI studies may be a biased sample of schizophrenia patients due to stringent exclusion criteria. Paranoia, anxiety or claustrophobia can also prevent patients from participating. Therefore, there is a dire need to develop an alternative imaging strategy.

Near infrared spectroscopy (NIRS) is a relatively new, non-invasive method that allows us to investigate functional brain activation patterns [41]–[43]. When light was found to be a sensitive tool for measuring the hemoglobin concentration and oxygenation in the blood [44], imaging of reflected light began to be widely used in studies of animals to examine the functional architecture of the cortex. NIRS uses the near infrared spectrum (700–1000 nm wavelength) to monitor changes in oxyhemoglobin and deoxyhemoglobin *in vivo*. Light from the near infrared spectrum can penetrate the skull and is absorbed mainly by oxy- and deoxyhemoglobin which have different absorption spectra. Changes in the oxy- and deoxyhemoglobin in the brain tissue can be calculated from the amount of absorbed near infrared light using the modified Lambert-Beer Law, as mutually exclusive windows of near infrared wavelengths are sensitive to oxy- and deoxyhemoglobin substrates [45]. Thus, NIRS measures cortical activation based on oxygenation of hemoglobin, similar to the fMRI BOLD response. Furthermore, NIRS provides reasonable spatial resolution and has advantages over fMRI including good temporal resolution, better motion tolerance, no noise, and increased comfort for the participants. Obesity and metal implant(s) are not impediments. These characteristics of NIRS suggest its role as a suitable alternative method of fMRI in psychiatric research. However, before NIRS can be used widely in psychiatric research, it should be validated against fMRI and other imaging methods. In Experiment 2, we used NIRS to examine the neural correlates of working memory in schizophrenia, using the same task as in Experiment 1.

To summarize, we used two functional neuroimaging methods to examine spatial working memory function in schizophrenic patients and healthy control subjects. We used a spatial delayed response task, which has been studied extensively in neurophysiological studies of working memory in nonhuman primates and thus provides a close animal analogue. With an event-related design, we aimed to observe cortical activation during correct vs. error trials in order to understand the neural correlates of remembering and forgetting. In addition, we examined qualitatively distinct types of error trials to further specify the behavioral and neural difference between errors that arise from a loss of mental representation versus those which arise from encoding an incorrect stimulus.

## Results

### Experiment 1: An event-related fMRI study of spatial working memory maintenance in schizophrenic and healthy control participants

Behavioral data show that healthy controls were more accurate than schizophrenia patients (mean = 86±9% and 75±9%, respectively, t_13_ = 2.39, p<0.05) on the spatial working memory task overall. To examine different types of errors in relation to brain activation, we divided total responses into two categories based on the confidence ratings; ‘confident’ for the ratings 1 and 2 and ‘not-confident’ for the ratings 4 and 5. We then examined the accuracy of ‘confident’ responses. 84% of healthy controls' total responses were rated as confident and among these, 92% were correct. Schizophrenia patients rated 86% of their total responses as confident, but only 77% of these were correct, producing more incorrect but confident responses compared with controls (χ^2^ = 17.41, p<0.001). This result suggests that schizophrenia patients may have encoded and maintained ‘wrong’ representations in working memory and as a result, produced incorrect but confident responses (i.e. “false memory” response).

**Figure 2:**
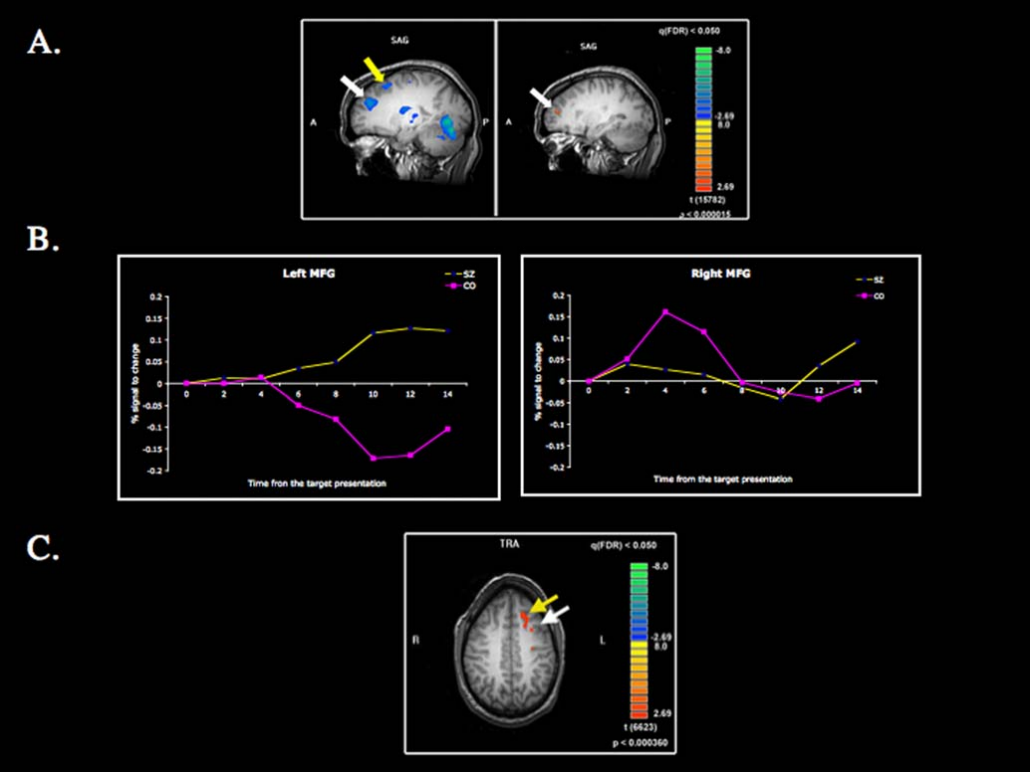
Activation maps of the delay-related BOLD data in the frontal cortex during the spatial WM task. (A) For correct trials, schizophrenic patients (SZ) showed increased activation in the middle frontal gyrus (white arrow) and the superior frontal gyrus (yellow arrow) in the left hemisphere whereas the control group (CO) showed increased activation in the middle frontal gyrus and the superior frontal gyrus in the right hemisphere. (B) This figure shows the % signal change during the spatial WM task in the right MFG and the left MFG for correct trials. In the left MFG, schizophrenic patients showed increased activation during maintenance. (C) Schizophrenic patients showed increased activation in the middle frontal gyrus and the superior frontal gyrus during “false memory” trials.

The delay-related (maintenance phase) BOLD activity of schizophrenia patients was compared with that of healthy controls on correct trials. Since we selected only the correct trials for this comparison, the behavioral performance is matched across the two groups. Table 1 displays the brain regions activated during spatial working memory maintenance in both groups. During the delay period, healthy control subjects showed increased activation in the right middle frontal gyrus (MFG) and the right superior frontal gyrus (SFG) (Figure 2). In addition, controls showed greater activation in the right posterior cingulate gyrus (PCG) and the right superior temporal gyrus (STG) compared with schizophrenia patients. Schizophrenia patients recruited a wider network at the same level of performance. One significant importance is that they activated several left hemisphere regions during spatial working memory maintenance. The patients showed a greater activation compared with the controls in the left MFG, the left inferior frontal gyrus (IFG), the left CG as well as the left inferior parietal lobule (IPL) and the left STG. In the right hemisphere, they showed increased activation in the anterior part of the right CG, the right insular, the right MFG and the right SFG. With respect to the right frontal activation in schizophrenic patients, the increased activity seems to occur later than in control subjects.

**Table 1:** Talairach sterotaxic coordinates for the peak of activated areas in the brain during the spatial working memory maintenance.

	*x*	*y*	*z*	*t*
Control>Schizophrenia				
Right middle frontal gyrus	41	38	15	2.6
Right superior frontal gyrus	25	46	15	3.0
Right posterior cingulate gyrus	13	−42	7	3.1
Right superior temporal gyrus	53	−3	−11	2.9
Schizophrenia>Control				
Right middle frontal gyrus	19	32	43	−2.7
Right superior frontal gyrus	16	12	48	−2.3
Left middle frontal gyrus	−39	3	48	−7.6
Left inferior frontal gyrus	−47	1	1	−7.7
Left medial frontal gyrus	−4	9	47	−2.6
Left superior temporal gyrus	−44	12	−12	−7.2
Left inferior parietal lobule	−34	−47	43	−7.0
Right cingulate gyrus	9	21	32	−3.5
Left cingulate gyrus	−7	−3	42	−3.8
Right insular	43	1	1	−6.0

To test whether schizophrenia patients indeed maintained mental representations during “false” memory trials, the delay-related BOLD activity for error trials with high confidence ratings was examined in schizophrenia patients (Figure 2). Similar analyses for healthy controls could not be performed due to the small number of such false memory trials. On false memory trials, schizophrenia patients activated left MFG, SFG, CG, and IFG and the right MFG; similar to the areas recruited during the correct working memory trials in schizophrenia patients. It is likely that schizophrenia patients maintained the internal representation of incorrect stimuli during these trials, resulting in active recruitment of a working memory network.

The right MFG was activated for both groups during the spatial working memory maintenance phase, but schizophrenia patients activated additional left hemisphere regions (left MFG and IFG). Clearly lateralized activation in the right PFC during spatial working memory was only observed in healthy controls. In addition, schizophrenia patients activated the same bilateral network on correct and false memory trials, suggesting that they maintained the representation of ‘wrong’ targets during those trials. This finding illustrates the utility of more fine-grained analysis of errors in specifying the potential causal factors leading to working memory deficits.

There are caveats. Age and education were not matched across groups. To address this issue, we compared a subset of controls (n = 5) who were age- and education- matched to schizophrenia patients and observed the identical pattern of results. Secondly, the sample size was small. These two problems are common in fMRI studies of psychiatric patients because many patients are unwilling to or unable to participate in a MRI experiment. Therefore, there is a need to develop and test an alternative imaging method that allows participation by a larger number of patients. We conducted an identical spatial working memory experiment using NIRS.

### Experiment 2: An event-related near infrared spectroscopy (NIRS) study of spatial working memory maintenance

Accuracy on the spatial delayed response task was compared between the two groups. Behavioral data showed a trend towards a group difference (accuracy; controls, 88±7%, schizophrenia patients, 80± 2%, t_21_ = 1.83, p = 0.08). We divided the responses according to the confidence ratings, as described above in the fMRI results section. Healthy controls rated 81% of their total responses as confident and of those, 91% were correct. Schizophrenic patients rated 74% of the total responses as confident, and 81% of these confident responses were correct. Patients produced more incorrect but confident responses compared with healthy controls (χ^2^ = 14.2, p<0.001), suggesting that they were prone to ‘false memory’ errors. These behavioral data replicate those observed in the fMRI experiment described in the previous section.

**Figure 3:**
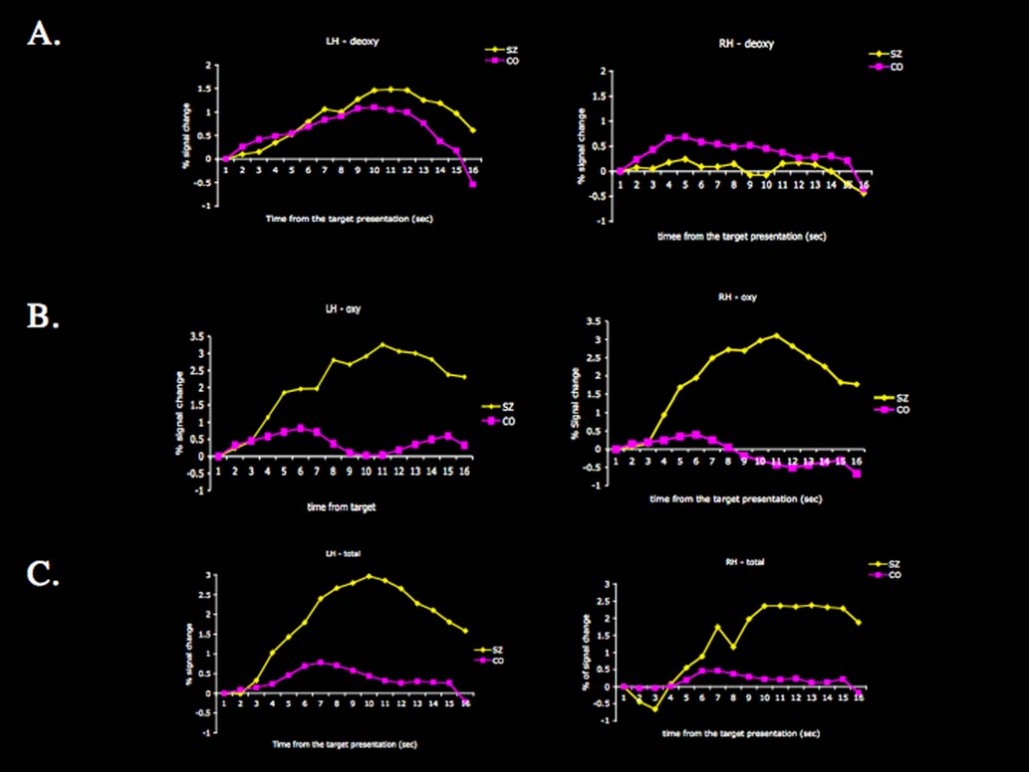
NIRS data comparing maintenance-related NIRS measurements between the control (CO) and schizophrenic (SZ) groups during the spatial WM task. (A) Deoxy-Hb data: schizophrenic patients showed greater activity in the left frontal cortex (see the left panel) and the healthy control subjects showed greater activation in the right frontal cortex (see the right panel) on correct trials. (B) Oxy-Hb data: schizophrenic patients showed greater activation in the bilateral frontal cortex on correct trials. (C) Total-Hb data: schizophrenic patients showed greater activation in the bilateral frontal cortex compared with the control subjects on correct trials.

To examine whether schizophrenia patients showed a differential activation pattern during spatial working memory maintenance, delay-related changes in NIRS data of patients were compared with that of controls on correct trials. Oxyhemoglobin (Oxy-Hb), deoxyhemoglobin (deoxy-Hb), and total hemoglobin (total-Hb) data were compared separately. Figure 3 presents time course graphs of peak activation for the oxy-Hb, deoxy-Hb and total-Hb in the frontal cortex. Healthy controls showed greater deoxy-Hb concentration in the right frontal cortex whereas schizophrenic patients showed greater deoxy-Hb concentration in the left frontal cortex. Schizophrenic patients showed greater activation in the bilateral frontal cortex as indicated by both oxy-Hb and total-Hb concentrations, but this pattern was more prominent in the left PFC.

Schizophrenic patients produced a significant number of confident but incorrect responses (‘false memory’ errors) during the spatial working memory task. The delay-related NIRS data for false memory errors was examined. A similar analysis in healthy controls could not be performed due to the minute number of false memory errors. Schizophrenic patients showed decreased oxy-Hb during the false memory trials in both hemispheres. For deoxy-Hb data, increased deoxy-Hb in the left frontal cortex and decreased deoxy-Hb in the superior part of the right frontal cortex were observed. Schizophrenic patients showed increased total-Hb in both hemispheres during the false memory trials indicating an increased activity of the frontal regions that accompany memory maintenance. Thus, it seems likely that incorrect target representation was maintained during the delay period on such trials.

## Discussion

We investigated the neural correlates of spatial working memory maintenance in schizophrenic patients and healthy controls using two functional neuroimaging methods: fMRI and NIRS. By using an event-related design and analyzing distinct types of correct and error trials, we aimed to elucidate the role of prefrontal activity in remembering and forgetting. We found that prefrontal activation was increased in both healthy controls and schizophrenic patients on correct memory trials but that the expected hemispheric specialization was greatly reduced in schizophrenic patients. While healthy subjects showed an increased activation of right hemisphere systems during the spatial working memory task, schizophrenic patients tended to show more bilateral or left hemisphere activity. This pattern cannot be due to differences in performance since only correct trials were included in this analysis. Thus, even when behavioral performance is matched, neural activation pattern shows a significant difference between schizophrenic and healthy subjects. In addition, by analyzing different types of errors, we observed that schizophrenia patients, but not healthy control subjects, made false memory errors where they were confident of their memory in spite of incorrect performance. On such error trials, schizophrenic patients showed increased prefrontal activation identical to that shown when they remembered correct locations.

### Prefrontal cortex and spatial working memory maintenance in schizophrenia

In this study, healthy controls showed the expected lateralized pattern [27] of activation of the right hemisphere networks during the spatial working memory task. However, this pattern was not observed in schizophrenia patients. The bilateral prefrontal activation of schizophrenia patients on correct trials suggests that a ‘simple’ model of hypofrontality or hyperfrontality cannot account for their working memory deficits. A recent meta-analysis indicates that there is a complex pattern of hyper- and hypo-activation in the regions associated with working memory in schizophrenia [46]. Walter et al. [36] also reported an absence of hemispheric asymmetry during verbal and spatial working memory but they did not observe hypo- or hyper-frontality. Since we compared only the correct trials of both groups, our finding is not an artifact of differential performance. Rather, different regions and a more diffuse network seem to be engaged during spatial working memory maintenance in schizophrenia.

Our results suggest that the cortical activation pattern in schizophrenia patients differs from that of controls, even when the resulting observed behaviors are indistinguishable from those of controls. More symmetrical pattern of activation during spatial working memory in schizophrenia may be the result of a compensatory mechanism for a dysfunctional right hemisphere (RH) in schizophrenia. Schizophrenia patients do *not* show a RH advantage for processing visuospatial information as controls do [36], [47]–[52]. RH dysfunction in schizophrenia may be due to reduced or absent brain asymmetry [49]–[52] and/or abnormal hemispheric interaction [53]. Reduced hemispheric asymmetry in schizophrenia may result in a diffuse activation pattern during a specific task, in contrast to a more selective activation of specialized network in controls. It has also been suggested that schizophrenia patients have abnormal cortico-cortical connectivity which may result in anomalous hemispheric specialization of functions [53]–[57].

Another possibility is that the bilateral activation observed may reflect compensatory or increased cognitive effort exerted by schizophrenia patients to achieve correct performance. Schizophrenic patients may have reduced working memory capacity compared with controls [58], [59], which means they may find the same task more difficult than do controls, and increasing task difficulty has been shown to result in bilateral activation pattern during a working memory task in healthy individuals [60], [61]. Thus, schizophrenia patients may increase effort to compensate for reduced working memory capacity to reach the same performance level as controls, resulting in a less lateralized prefrontal activation pattern.

Our results also showed that the same brain regions were activated for “false” memory trials where errors were coupled with a high degree of confidence. On these trials, schizophrenia patients were very certain that they remembered the targets even though they were incorrect. This finding suggests that schizophrenia patients may have encoded ‘wrong’ targets and maintained the representations with corresponding frontal cortical activation on these false memory trials. Therefore, if patients make a large number of such errors, they may show normal or even hyperfrontal activation during a working memory task in spite of high error rates. The finding of robust activation during false memory trials may partly account for the inconsistent findings of previous studies that have reported hyper and hypofrontality.

### Using NIRS for psychiatric research

During the spatial working memory task, healthy controls showed greater activation in the right frontal cortex compared with schizophrenia patients, whereas schizophrenia patients showed greater activation in the left frontal cortex in deoxy-Hb. In the oxy-Hb and total-Hb, schizophrenic patients showed hyperactivation compared with controls in bilateral frontal cortex. These suggest that in healthy controls, right frontal cortex clearly supports spatial working memory; but in schizophrenia patients there is additional activation of the left frontal regions, even when the two groups are matched for behavioral performance.

Our NIRS results diverge slightly from previous studies of schizophrenia using NIRS. We observed increased activation of PFC from oxy-Hb and total-Hb in schizophrenia patients. Additionally, we observed that the deoxy-Hb data were similar to fMRI data. Previous NIRS studies of schizophrenia have shown hypoactivation in the prefrontal cortex especially in oxy-Hb, accompanied with poorer behavioral performance; but the degree of hypoactivation has varied depending on the specific task even within the same study [62]–[64]. For instance, Shiba et al. [62] observed smaller oxy-Hb increases in schizophrenic patients during a random-number generation task compared with controls, but not in a sequential learning task. It is possible that functional abnormalities in the prefrontal cortex may be task-dependent in schizophrenic patients. In addition, we separated correct from incorrect trials to relate brain activation to performance. Therefore, the brain-behavior relationship we tracked in our study is more specific than has been observed in previous studies.

We reported all 3 NIRS measurements, each showing some evidence of frontal cortical abnormalities in schizophrenia. Some researchers have argued that among the three Hb measures, deoxy-Hb may be the most accurate indicator of cortical activation because increased oxy-Hb may indicate a change in blood pressure or an increase in skin blood volume [65]. Deoxy-Hb has also been shown to be associated with the BOLD contrast of fMRI [65], [66]. However, other studies suggest that oxy-Hb may be better than deoxy-Hb for estimating cortical activation [67]. Some studies have reported that oxy-Hb level is more closely related to the BOLD signal of fMRI than deoxy-Hb [68]. Moreover, some report that there are changes in oxy-Hb during cognitive tasks, but not in deoxy-Hb [68], [69], although this could be due to the fact that oxy-Hb signals are much larger and therefore easier to detect [70], [71]. Thus, there is still disagreement as to which parameters may best reflect cortical function in humans. A practical solution would be to report all three measures.

Overall, the results from the NIRS experiment are in agreement with those obtained from the fMRI experiment in our study and suggest that NIRS is a viable, alternative method.

### Summary

The findings of this study suggest that even with a very simple paradigm such as the delayed-response task, one must carefully consider the components of working memory in relation to brain activation patterns if we are to understand potential individual differences. Most functional imaging studies of working memory do not distinguish among different types of errors. However, our results suggest that such analyses provide novel insight into the etiology of working memory deficits in schizophrenia.

We used two imaging techniques in this study. We found that NIRS and fMRI yield comparable data and therefore NIRS could be used as a viable alternative method for situations that are incompatible with MRI. For psychiatric neuroimaging, NIRS could allow increased patient participation.

There are, however, caveats. The sample size in our fMRI experiment was small. This is offset by the NIRS experiment with a larger sample, but it would be important to replicate these results in a future study. All patients were taking atypical antipsychotic drugs. Therefore we cannot rule out medication effects, but it has been shown that first episode schizophrenia patients [72], unmedicated schizophrenia patients [73] and unmedicated, healthy first-degree relatives of schizophrenia patients [3], [5] show spatial working memory deficits. While a recent study suggests that risperidone may worsen working memory in first episode patients, the same study also reported that the working memory deficit is already present before medication is administered [74]. Although it seems unlikely that the current findings stem from medication effects, a future study should address it systematically. Lastly, it is important to note that the event-related design and data analyses that segregated correct from error trials allowed us observe the neural correlates of remembering and forgetting, regardless of the overall performance. On correct working memory trials, schizophrenia patients were remembering correctly even if their overall error rates were greater than that of controls.

To summarize, we observed a reduced functional asymmetry of prefrontal cortex during a spatial working memory task in schizophrenia patients even when the behavioral performance was matched to that of controls who showed a clear right prefrontal activation pattern during the same working memory task. We also found an increased rate of false memory errors in schizophrenia patients, suggesting problems in encoding.

## Materials and Methods

### Experiment 1: An event-related fMRI study of spatial working memory maintenance in schizophrenic and healthy control participants

#### Participants

Eight schizophrenic outpatients were recruited from the Vanderbilt Psychiatric Hospital. They met the DSM-IV [75] criteria for schizophrenia or schizoaffective disorder based on clinical interviews and chart reviews. Exclusion criteria for schizophrenic subjects included past or current substance abuse, brain injury, neurological disease and medical illnesses known to affect brain function. Clinical symptoms were evaluated with the Brief Psychiatric Rating Scale (BPRS) [76]. All patients were taking atypical antipsychotic drugs (clozapine, risperidone, or olanzapine). Seven healthy control participants were recruited through advertisements. Exclusion criteria for control subjects were past or present DSM-IV Axis I or II disorder, a family history of psychotic illness in their first- or second-degree relatives, neurological disorder and any illness known to affect brain function. All participants were right-handed. The demographic information is presented in Table 2. Written informed consent was obtained from all participants after they were given a complete description of the study. The Vanderbilt University Institutional Review Board (IRB) approved the protocol and consent procedure. All participants were paid.

**Table 2:** Demographic information of participants

Experiment 1 fMRI				
	Schizophrenia	Control	Statistical Test	
Age	34.9 (9.3)	25 (4.5)	t_13_ = −2.43	p<.05
Education (years)	12.8 (1.2)	15.1 (2.3)	t_13_ = 2.4	p<.05
Gender (M/F)	5/3	4/3	χ^2^ = .04	NS
Handedness (R/L)	8	7		
BPRS	19.5 (4.4)	NA		

Experiment 2 NIRS				
	Schizophrenia	Control	Statistical Test	
Age	34.7 (8.0)	36.6 (6.4)	t_21_ = .58	NS
Education (years)	13.0 (1.0)	14.1 (2.4)	t_21_ = .58	NS
Gender (M/F)	9/4	7/4	χ^2^ = 0.08	NS
Handedness (R/L)	10/3	11/0	χ^2^ = 2.65	NS
BPRS	12.0 (8.5)	NA		

#### Spatial working memory (WM) Task

To examine maintenance-related brain activation, participants performed the spatial delayed response task (spatial DRT, Figure 1) adopted from Leung et al. [27]. Each trial began with presenting a fixation point for 1s. Then 3 targets (identical black circles) were flashed sequentially on a gray background, each in a different location (750 ms each) with the inter-stimulus interval of 250 ms. After a 12s delay, a probe was presented for 3s. Participants were asked to decide whether the probe was at one of the three target locations by pressing their right thumb (YES) or right index finger (NO). Immediately after the decision, participants indicated the confidence level for their response on a 5-point rating scale that ranged from 1 (most confident) to 5 (least confident) by pressing a button corresponding to the 5 digits; “1” corresponded to the right thumb, “2” to the right index finger and so on. An inter-trial interval (ITI) of 8.25s followed the confidence rating before a new trial began. There were seven runs containing 14 trials per run. Participants made their responses (the remembered location of the target and the confidence level of their response) using their dominant hand (i.e., right hand).

#### Image acquisition

All brain images were collected on a 3 Tesla whole-body GE Signa MRI system with a birdcage head coil at the Vanderbilt University Medical Center. 19 T1-weighted anatomical images parallel to the anterior and posterior commissures (AC-PC) were acquired, along with T2*-weighted functional images parallel to the AC-PC line for BOLD-based images (gradient echo planar imaging sequence, TR = 2000ms, TE = 35ms, flip angle = 90°, matrix = 64×64, slice thickness = 5mm, slice gap = 1mm, FOV = 24×24cm). High-resolution T1-weighted anatomical volumes were also acquired with a magnetization-prepared 3D SPGR imaging sequence. Stimuli were presented through MR-compatible LCD goggles (VisualStim XGA Resonance Technology).

#### fMRI data analysis

Imaging data were preprocessed and analyzed using BrainVoyager 4.9 and QX (Brain Innovation, Maastricht, Holland). Anatomical volumes were transformed into a common stereotactic space [77]. Functional volumes for each subject were aligned to the transformed anatomical volumes, thereby transforming the functional data into a common brain space across participants. Data pre-processing for functional volumes included image realignment, three-dimensional motion correction, linear de-trending, temporal frequency filtering with high pass filter, and spatial smoothing with a 4-mm Gaussian kernel (full width at half-maximum). The statistical analysis was based on the application of the multi-study general linear model (GLM) to time-series of task-related function volumes. The GLM allows the correlation of predictor variables with the recorded activation data (criterion variables) across scanning sessions on grouped datasets. The GLM in BrainVoyager with predictors of interest (i.e., correct vs. error trials determined from the behavioral data) was applied for the individual Z-normalized volume time courses to investigate the delay-related brain activity. The overall model of fit was assessed by an F-statistic. Significant differences among the conditions (e.g., correct vs. error) were assessed with contrast (*t*) maps. Obtained *p* values were corrected for multiple comparisons with False Discovery Rate (FDR) of .05. The FDR controls the expected proportion of false positives among suprathreshold voxels, instead of controlling for the change in any false positives as Bonferroni correction method does [78], [79].

### Experiment 2: An event-related Near Infrared Spectroscopy (NIRS) study of spatial working memory maintenance

#### Participants

Thirteen right-handed schizophrenic subjects (8 of them had participated in the fMRI experiment) and 11 matched healthy controls participated in the study. Recruitment of participants, and exclusion/inclusion criteria for the patients and healthy controls were identical as those in Experiment 1 (see Table 2 for demographic information). All patients were taking atypical antipsychotic drugs (clozapine, risperidone, and olanzapine). All participants were provided a complete description of the study and gave written informed consent. The Vanderbilt University IRB approved the study protocol and consent procedure and all participants were compensated.

#### Spatial WM Task

The same spatial WM task used in Experiment 1 was administered.

#### NIRS Measurement

NIRS was performed using a 24-channel (maximum) 780–830 nm spectrometer (ETG-100 system; Hitachi Medical Corp.), composed of emitter-detector pairs. Each emitter was composed of two continuous laser diodes (3mW± 0.15 mW on ‘high’ power) with different wavelengths (780±20 and 830±20 nm) that were amplitude modulated (0.6 and 1.5 Khz). The distance between the pair of emitter and detector probe was 30 mm, which produced a light penetration close to 20 mm. Signals were acquired at a sample rate of 10 Hz from 22 cortical regions on the bilateral prefrontal cortex using the 3×5 probe holder and the corresponding optodes. The signal was amplified, demodulated, and then digitized.

#### Anatomical Localization

Probes were placed on the forehead according to the international 10–20 system of electrode placement used for EEG and ERP. The middle vertical band of optodes was placed along the z (midline) axis extending from the Fp position ventrally (just superior and horizontal to the Obicularis oculi muscles) towards a caudal position proximal to the Fz position along the Frontalis muscles. However, unlike EEG electrode placement that uses relative (10–20% distance from nasion and inion) place of electrodes in individual skulls, the NIRS optodes were placed in a fixed holder that cannot be stretched or compressed for individual variation in skull measurements. Therefore, some anatomical variation inevitably exists among individuals. However, this method assures a high level of standardization across participants with the right hemisphere probes covering areas Fp2, F4 and F8 and the left hemisphere probes covering area Fp1, F5 and F7.

#### NIRS data analysis

NIRS data were analyzed using Matlab (The Mathworks) and Brain Voyager-QX. First, a temporal filter was applied to remove any artifact due to respiration and cardiac variation using a bandpass filter with a range 0.01–0.5 Hz. After temporal downsampling (from 10–1 Hz.), NIRS data were converted to the measurement of oxyhemoglobin (oxy-Hb), deoxyhemoglobin (deoxy-Hb), and total hemoglobin (total-Hb) levels according to the modified Beer-Lambert Law [45]. These data were converted into a format compatible with Brain Voyager QX using an in-house program after an addition of a constant (500) to each measurement (required to make NIRS data compatible with raw signal data colleted in magnetic resonance research). This allowed us to analyze NIRS data in exactly the same way as in Experiment 1. Data pre-processing included linear de-trending to remove overall linear drifts. The GLM was used to compare whether schizophrenic subjects showed a different pattern of brain activation compared to healthy control subjects, focusing on the delay period of the spatial working task. Thus, the significance of predictors of interest was studied using an F-test followed by paired comparisons. The FDR of .05 was used to correct for multiple comparisons.
